# MicroRNA-22 Regulates the Pro-inflammatory Responses and M1 Polarization of Macrophages by Targeting GLUT1 and 4-1BBL

**DOI:** 10.1155/2023/2457006

**Published:** 2023-07-10

**Authors:** Young Jun Kang

**Affiliations:** Molecular Medicine Research Institute, Sunnyvale, CA 94085, USA

## Abstract

Many microRNAs (miRNAs) are selectively expressed in mammalian immune cells and have been linked to immune responses in host defense and autoimmune disease. In macrophages, miRNAs regulate cell metabolism by repressing the expression of genes such as transcription factors, enzymes, and metabolism-related molecules, as well as the expression of genes that impact inflammatory responses and phenotype determination. Previous studies showed that miR-22 plays a role in a variety of biological processes, such as cancer cell growth, cell survival, and cell expansion. In CD4 + *T* cells of inflammatory bowel disease patients, miR-22 is upregulated and regulates inflammasome-mediated responses. However, it has not yet been determined how miR-22 contributes to the activation of innate immune cells. In this study, we identified a mechanism of toll-like receptors- (TLR-) dependent miR-22 induction that regulates the downstream signaling pathway linking inflammatory responses and macrophage polarization. MiR-22 is induced via TLR-signaling, which regulates the induction of *Slc2a1* (glucose transporter 1 and Glut1) and *Tnfsf9* (tumor necrosis factor 9, 4-1BB ligand, and 4-1BBL) mRNAs that contribute to sustained inflammatory responses and the polarization of macrophages. Our observations support further efforts to explore a potential therapeutic strategy using miR-22 for the modulation of excessive macrophage activation for the treatment of inflammatory diseases.

## 1. Introduction

Macrophages play a critical role in inflammation, tissue repair, host defense against microbial infection, and tumor surveillance. Previous studies have helped elucidate the mechanism that regulates the polarization of macrophages, a process that underlies the specialized functions of macrophages in different microenvironments.

Macrophage polarization is a crucial event for the induction of host defense, tissue repair, and homeostasis maintenance, referring to the process by which macrophages produce distinct functional phenotypes in response to different stimuli and signals [1–3]. Macrophages can be polarized into pro-inflammatory M1 (classically activated) and anti-inflammatory M2 (alternatively activated) phenotypes. Polarized macrophages differ in their cell surface markers, secreted cytokines, and biological functions. Following the activation by lipopolysaccharide (LPS) or Th1 cytokines, such as IFN-*γ* and TNF-*α*, macrophages are polarized into M1 macrophages that express CD80, CD86, iNOS, and MHC-II on the surface and produce inflammatory cytokine such as TNF-*α*, IL-1*β*, and IL-6. In contrast, M2 polarization is induced by IL-4 and/or IL-13. M2 macrophages express the surface markers like CD206, CD163, Fizz-1, and Ym-1, and the production of anti-inflammatory cytokines, such as IL-10 and TGF-*β*, is increased in M2 macrophages. M1 macrophages can initiate and sustain inflammatory responses, releasing pro-inflammatory cytokines, and recruiting other immune cells into the site of inflammation, while M2 macrophages plays a role in the resolution of inflammation, phagocytosis of apoptotic cells, secretion of anti-inflammatory mediators, and wound healing.

Phenotypic and functional changes of macrophages is accompanied by the switches in cell metabolism. Specifically, M1 macrophages mainly rely on glycolysis and exhibit impairment of Krebs cycle and mitochondrial oxidative phosphorylation (OXPHOS), whereas M2 cells are more dependent on mitochondrial OXPHOS. Moreover, pro- and anti-inflammatory macrophages regulate the specific lipid metabolism for the responses as fatty acid synthesis is crucial in M1 macrophages and fatty acid oxidation plays an essential role in M2 responses amino acids and affect their responses [4–7]. Additionally, lactate dehydrogenases are critical enzymes in glycolysis and cell metabolism is related to the cellular metabolic state, aerobic or anaerobic direction of glycolysis, and activation status [8, 9].

MicroRNAs (miRNAs) are an evolutionally conserved group of short (∼ 22–24 nucleotides) noncoding RNAs that regulate the expression of many genes by targeting the 3′ UTR of mRNAs in most cases [10, 11]. However, in some cases, miRNAs interact with other regions, including the 5′ UTR, coding sequence, and gene promoters. The binding of miRNAs to 5′ UTR and coding regions have silencing effects on gene expression while miRNA interaction with promoter region has been reported to induce transcription [12]. However, the underlying mechanism and the functional significance of such mode of interaction are not fully understood. Many miRNAs are selectively expressed in mammalian immune cells, with some being linked to immune responses in host defense and autoimmune disease [13–17]. MiRNAs regulate macrophage metabolism by repressing the expression of genes such as transcription factors, enzymes, and metabolism-related molecules, further impacting inflammatory responses and phenotype determination [13, 18]. In light of these observations, miRNAs are thought to play a crucial role in host defense and inflammatory diseases [19–21].

MiR-22 is involved in a variety of biological processes such as cancer cell growth, cell survival in the skin, and keratinocyte progenitor cell expansion [22–28]. Additionally, miR-22 is upregulated in CD4 + *T* cells of patients with inflammatory bowel disease [29]. However, the role of miR-22 in the activation and the metabolic regulation associated with phenotype determination in macrophages has not been fully elucidated.

In this study, we found that miR-22 is induced by toll-like receptors (TLR) signaling and regulates the pro-inflammatory responses in macrophages. MiR-22 suppresses the expression of *Slc2a1* and *Tnfsf9* mRNAs, thereby regulating the inflammatory responses and metabolic reprograming of macrophages. Our observations support further exploration of potential therapeutic strategies using miR-22 for the modulation of macrophage functional responses in diseases characterized by excessive immune-mediated inflammation.

## 2. Materials and Methods

### 2.1. Reagents

We obtained recombinant murine IFN-*γ*, murine IL-4, and murine M-CSF from Peprotech (Cranbury, NJ, USA), LPS (*Escherichia coli* 0111:B4) from List Biological Laboratories (Campbell, CA, USA), and Pam3CysSerLys4 (Pam3Cys), poly I:C, imiquimod (IMQ), and CpG DNA from Invivogen (San Diego, CA, USA). Bay 11-7082, SB203580, SP600125, and PD98059 were purchased from Calbiochem (Burlington, MA, USA).

### 2.2. Animals

Sex-matched 8–12-week old male or female C57BL/6 mice were purchased from Jackson Laboratory (Bar Harbor, ME, USA). Femur and tibia were obtained from mice for bone marrow cell culture. Animal experiments complied with the regulations of the Institutional Animal Care and Use Committee in accordance with guidelines of the Association for the Assessment and Accreditation of Laboratory Animal Care.

### 2.3. Cell Culture

RAW 264.7 mouse macrophage cell line and HEK293T cell line were cultured in DMEM (Invitrogen, Waltham, MA, USA) supplemented with 10% FBS and antibiotics. Bone marrow cells were obtained from femur and tibia of mice and cultured in DMEM supplemented with 10% FBS, antibiotics, and 10 ng/ml M-CSF for 7 days.

RAW 264.7 (5 × 10^5^/ml) or BMDMs (1 × 10^6^/ml) were treated with LPS (100 ng/ml), Pam3Cys (5 *μ*g/ml), poly I:C (25 *μ*g/ml), IMQ (2 *μ*g/ml), CpG DNA (5 *μ*g/ml), LPS + IFN-*γ* (100 ng/ml), or IL-4 (10 ng/ml).

### 2.4. MicroRNA Plasmids, Lentivirus Preparation, and miR-22 Macrophage Generation

Lentiviral plasmids expressing the control (empty), miR-22 mimic, or miR-22 inhibitor sequences were obtained from Biosettia (San Diego, CA, USA). Lentiviruses were prepared in 293 *T* cells by cotransfection of miRNA-encoding plasmids and helper plasmids such as pRSV-REV, pMDLg, and pVSV-G (Addgene, Watertown, MA, USA). To generate control, miR-22-overexpressing, or miR-22 inhibitor-expressing macrophage cells, RAW 264.7 were infected with lentiviruses and further incubated with puromycin.

### 2.5. Dual-Luciferase Reporter Assay

HEK293T cells were cotransfected with either control or miR-22 plasmids, and wild-type (WT) pGLO-*Slc2a1*-3′UTR or mutant (MUT) pGLO-*Slc2a1*-3′UTR, or WT pGLO-*tnfsf9*-3′UTR or MUT pGLO-*tnfsf9*-3′UTR using Lipofectamine 2000 according to the manufacturer's protocols. Cell lysates were obtained after 48 hr of transfection and luciferase activities were analyzed using the Dual-Luciferase Reporter Assay system (Promega Corporation, Madison, WI, USA). A plasmid constitutively expressing *Renilla* luciferase (pRL-TK *Renilla* luciferase) was cotransfected as an internal control.

### 2.6. Reverse Transcription and Quantitative PCR

Total RNA was isolated using RNeasy mini or miRNeasy mini kits (Qiagen, Germantown, MD, USA) depending on the experiment. To measure the expression of miR-22, cDNA was prepared using TaqMan MicroRNA Reverse Transcription kit (Thermo Fisher Scientific, Waltham, MA, USA) and miR-22 expression was analyzed using a TaqMan MicroRNA Assay, according to the manufacturer's protocol using TaqMan miRNA Assays Mouse sequence-specific primers (Thermo Fisher Scientific). RNU48 level was measured as an internal control.

Expression of inflammation-related genes such as *Il6*, *Il10*, *Il23*, *Tgfb*, *Tnf*, *Tnfsf9*, *Nos2*, *Arg1*, *Cxcl10*, *Fizz1*, *Ym1*, *Egr2*, and *Mrc1*, and cell metabolism genes such as *Slc2a1*, *Acaca*, *Acly*, and *Fasn* was measured. To this end, cDNA was prepared using a SuperScript V Reverse Transcriptase Kit (Thermo Fisher Scientific), and qPCR was performed using PowerUp SYBR Green Master Mix (Thermo Fisher Scientific). Gene expression was calculated by normalizing to *Gapdh* mRNA levels. Primer sequences are shown in *Supplementary 1*.

### 2.7. Immunoblotting

For immunoblotting analyses, the whole cell lysates were prepared in RIPA lysis buffer (pH 7.4, 150 mM NaCl, 50 mM tris, 0.5% sodium deoxycholate, 0.1% SDS, and 1% NP-40) containing protease inhibitors. Cell lysates were separated on SDS–PAGE and transferred to PVDF membrane for immunoblotting using GLUT1 (Sigma, St. Louis, MO, USA), 4-1BBL (R&D systems, Minneapolis, MN, USA), phospho-p38 MAPK (Thr180/Tyr182), phospho-JNK (Thr183/Tyr185), phospho-ERK (Thr202/Tyr204), and I*κ*B-*α* antibodies (Cell Signaling Technology, Danvers, MA, USA). GAPDH levels were detected as a loading control using anti-GAPDH antibody (Santa Cruz Biotechnology, Dallas, TX, USA). The proteins were detected by chemiluminescence using ECL substrates (ThermoFisher) [30].

### 2.8. Extracellular Flux Analysis

Metabolic changes were monitored by an extracellular flux analyzer XFe96 (Seahorse Bioscience). Cells were resuspended in XF Base Media (DMEM, Seahorse Bioscience) supplemented with 2 mM glutamine, and seeded in a Seahorse Bioscience 24-well plate. After 3 hr, cells were treated with medium, LPS + IFN-*γ* or IL-4, and incubated for 24 hr at 37°C in an atmosphere of 5% CO_2_. To measure the extracellular acidification rate (ECAR, glycolysis indication), rotenone plus antimycin A (Rot/AA, 1 *μ*M each), and deoxy-2-glucose (2-DG, 80 mM) were added sequentially. For measuring the oxygen consumption rate (OCR, mitochondrial respiration indication), cells were sequentially treated with oligomycin (1 *μ*M), N^5^,N^6^-bis (2-Fluorophenyl)- (1,2,5) oxadiazolo (3,4-b) pyrazine-5,6-diamine (BAM15, 2 mM), to cause mitochondrial uncoupling, and rotenone plus antimycin A (Rot/AA, 1 *μ*M each). All chemicals were purchased from Calbiochem (Burlington, MA, USA).

### 2.9. Cytokine Measurements

Culture supernatants from LPS-treated macrophages were collected at the indicated times, and concentrations of TNF and IL-6 were measured by ELISA kits following the manufacturer's protocol (Thermo Fisher Scientific). Release of inflammatory cytokine proteins was compared with mRNA induction of *Tnf* and *Il6* genes [31].

### 2.10. Intracellular Triglyceride Accumulation

Triglyceride levels in macrophages were quantified using a colorimetric Triglyceride Assay Kit (Abcam, Waltham, MA, USA) according to the manufacturer's protocol [32].

### 2.11. Statistical Analysis

Statistical significance was analyzed by one-way ANOVA followed by Tukey's post hoc multiple comparison test or by unpaired *t*-test using Prism software (version 9, GraphPad, San Diego, CA).

## 3. Results

### 3.1. Induction of miR-22 in LPS-treated Macrophages

**Figure 1:**
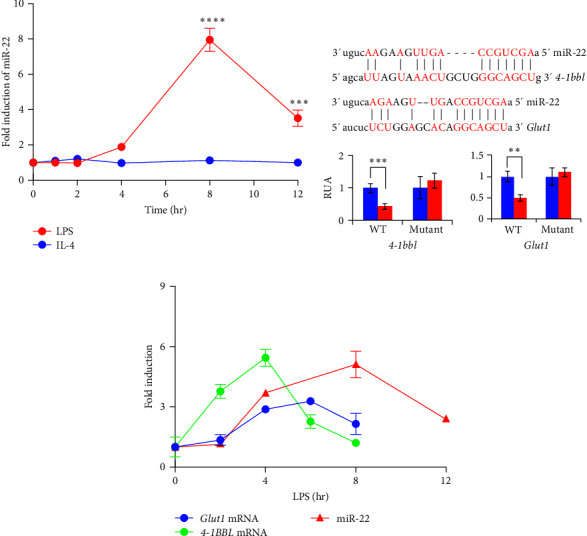
miR-22 is induced in BMDMs and targets GLUT1 and 4-1BBL. (a) BMDMs were treated with LPS or IL-4, and miR-22 levels were analyzed by qPCR. (b) HEK 293 *T* cells were transfected with luciferase reporter plasmid containing WT or MUT 3′-UTR of *Slc2a1* or *tnfsf9* with control (empty, miR-C) or miR-22 plasmid. (c) BMDMs were treated with LPS, and RNA samples were prepared at the indicated times and the induction of miR-22 and *Slc2a1* or *tnfsf9* mRNAs was analyzed by qPCR. Data are shown as mean ± SD. *N* = 3-4,  ^*∗∗*^*p* < 0.01,  ^*∗∗∗*^*p* < 0.005, and  ^*∗∗∗∗*^*p* < 0.001; Student *t* test. Result shown is representative of 2-3 independent experiments.

To explore the role of miR-22 in macrophages, we investigated whether the activation of macrophages induces the expression of miR-22. We observed that LPS stimulation increased endogenous miR-22 levels, whereas IL-4 did not, indicating that miR-22 is involved in TLR4 signaling (Figure 1(a)). Next, we determined the target mRNAs of miR-22 using bioinformatics tools such as miRbase [33] and TargetScan [34]. We selected 4-1BBL and GLUT1 as candidate genes and tested their candidacy as targets of miR-22 using plasmids encoding the 3′UTR of *Slc2a1* and *tnfsf9* mRNAs containing a putative miR-22-binding site. Using a luciferase assay, we found that miR-22 suppressed the expression of luciferase reporters fuzed with the wild-type *Slc2a1* or *tnfsf9* 3′ UTR but not that of mutant plasmids (Figure 1(b)).

The kinetics of miR-22 and *tnfsf9* or *Slc2a1* expression in LPS-treated macrophages were examined. The mRNA levels *tnfsf9* and *Slc2a1* increased first after LPS treatment, followed by the induction of miR-22 several hours later, leading to the subsequent decrease of *tnfsf9* and *Slc2a1*, indicating that miR-22 reduces the expression of 4-1BBL and GLUT1 at the late phase of macrophage activation (Figure 1(c)).

### 3.2. Overexpression of miR-22 Reduces the Expression of 4-1BBL and GLUT1 in Macrophages

**Figure 2:**
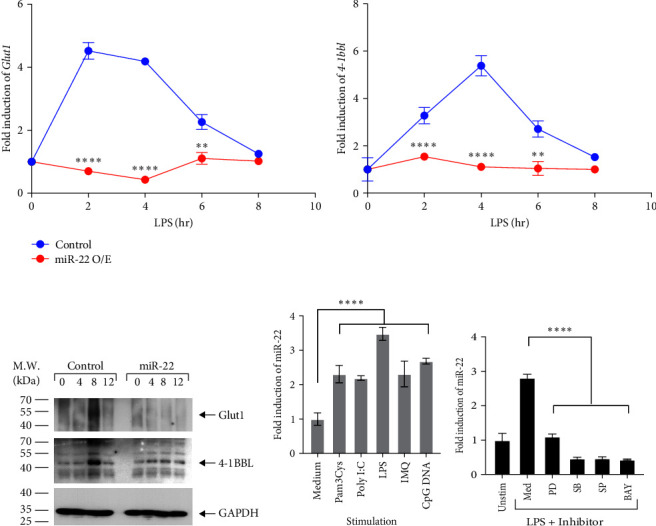
miR-22 represses the expression of *Slc2a1* and *tnfsf9* and induced via TLR-mediated signaling pathways. (a and b) Control or miR-22 O/E RAW 264.7 cells were treated with LPS. (a) *Slc2a1* or *tnfsf9* mRNA levels were analyzed by qPCR. (b) Protein levels were determined by immunoblotting using anti-GLUT1 or 4-1BBL Abs. GAPDH levels were detected as an internal control. (c) BMDMs were treated with medium or TLR ligands for 4 hr, and miR-22 levels were measured by qPCR. (d) BMDMs were incubated with medium, PD98059 (PD, 10 *μ*M), SB203580 (SB, 20 *μ*M), SP600125 (SP, 20 *μ*M), or Bay11–7082 (BAY, 10 *μ*M) for 1 hr followed by LPS stimulation. After 4 hr, induction of miR-22 was analyzed by qPCR. Unstim, unstimulated. Data are shown as mean ± SD. *N* = 4,  ^*∗*^*p* < 0.05,  ^*∗∗*^*p* < 0.01, and  ^*∗∗∗∗*^*p* < 0.001; ANOVA test or Student *t* test. Result shown is representative of three independent experiments.

We further investigated the role of miR-22 in regulating the expression of 4-1BBL and GLUT1 in macrophages. To this end, RAW 264.7 macrophages were infected with control (empty) or miR-22-encoding lentiviruses to generate control or miR-22-overexpressing (O/E) cells. The levels of tnfsf9 or Slc2a1 mRNAs increased after LPS stimulation in control macrophages, whereas miR-22-O/E significantly delayed or reduced the induction of mRNAs (Figure 2(a)). The protein levels of GLUT1 and 4-1BBL were analyzed by immunoblotting after 0, 4, 8, and 12 hr of LPS stimulation. LPS-induced GLUT1 expression was detected at 8 hr in control macrophages, but not detected in miR-22-overexpressing cells. Additionally, 4-1BBL expression peaked after 4 hr of LPS treatment in control macrophages; however, overexpression of miR-22 reduced 4-1BBL expression (Figure 2(b)). Collectively, our data indicate that the overexpression of miR-22 reduces the expression of GLUT1 and 4-1BBL, confirming that GLUT1 and 4-1BBL are the targets of miR-22.

### 3.3. Induction of miR-22 Is Mediated by TLR Signaling in Macrophages

Next, we examined whether miR-22 induction is dependent on TLR signaling in macrophages. To test this, macrophages from C57BL/6 mice were treated with Pam3Cys, poly I:C, LPS, IMQ, or CpG DNA, which are the ligands of TLR2, TLR3, TLR4, TLR7, and TLR9, respectively. Treatment with TLR ligands induced the expression of miR-22 (Figure 2(c)), whereas IL-4 did not (Figure 1(a)), supporting the role of TLR signaling in miR-22 induction in macrophages. Furthermore, we observed that miR-22 induction was regulated by the signaling pathways associated with macrophage activation such as NF-*κ*B and MAPKs. BMDMs were incubated with PD98059, SB203580, SP600125, or Bay11-7082 to inhibit the activation of MEK → ERK, p38, JNK, or NF-*κ*B, respectively. Compared with untreated cells, LPS-induced expression of miR-22 was significantly reduced by specific inhibitors (Figure 2(d)). Collectively, our data suggest that TLR signaling induces the expression of miR-22 via NF-*κ*B and MAPK signaling pathways in macrophages.

### 3.4. miR-22 Regulates the Pro-Inflammatory Responses and M1 Polarization of Macrophages

**Figure 3:**
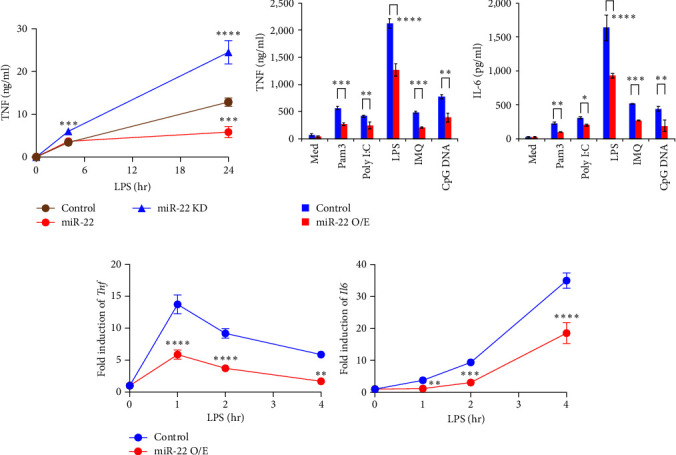
miR-22 regulates the pro-inflammatory responses. (a) Control, miR-22 O/E, or miR-22-Inh RAW 264.7 cells were stimulated with LPS, and TNF levels were measured. (b) Control or miR-22 O/E RAW 264.7 cells were stimulated with medium or TLR ligands for 24 hr, and TNF and IL-6 concentrations were measured by ELISA. (c) Induction of *Tnf* or *Il6* mRNAs in LPS-treated control or miR-22 O/E RAW 264.7 cells was determined by qPCR. Data are shown as mean ± SD. *N* = 4,  ^*∗*^*p* < 0.05,  ^*∗∗*^*p* < 0.01,  ^*∗∗∗*^*p* < 0.005, and  ^*∗∗∗∗*^*p* < 0.001; Student *t* test (a–c). Result shown is representative of at least three independent experiments.

We further investigated the role of miR-22 in the regulation of inflammatory responses in macrophages. To this end, control, miR-22-O/E, or miR-22 inhibitor-expressing (miR-22-Inh) macrophages were treated with LPS, and TNF levels were measured. LPS-induced TNF production was comparable during the first 4 hr after stimulation but was not sustained in miR-22-O/E cells compared with control. In contrast, inhibition of miR-22 increased TNF production, indicating that miR-22 regulates the pro-inflammatory responses in macrophages (Figure 3(a)). Additionally, production and gene expression of inflammatory cytokines such as TNF and IL-6 by various TLR ligands were significantly reduced in miR-22-O/E compared with control cells (Figure 3(b) and 3(c)).

**Figure 4:**
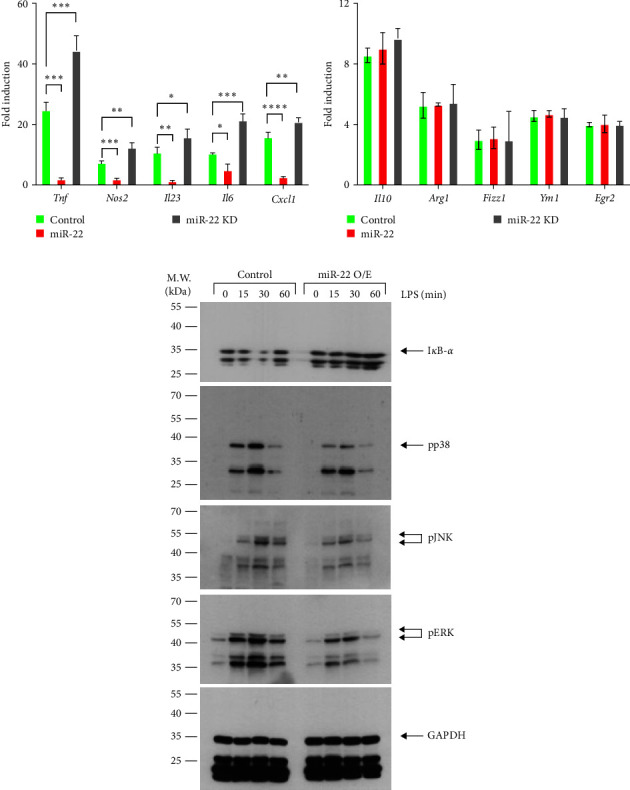
miR-22 regulates the M1 polarization by regulating the signaling pathways. (a and b) Control, miR-22 O/E, or miR-22-Inh RAW 264.7 cells were incubated with LPS + IFN-*γ* or IL-4 for 24 hr, and the expression of M1 (a) or M2 (b) genes was analyzed by qPCR. (c) Control or miR-22 O/E RAW 264.7 cells were treated with LPS, and the degradation of I*κ*B-*α* and the phosphorylation of p38 (pp38), JNK (pJNK), and ERK (pERK) by immunoblotting. GAPDH level was detected as a loading control. Data are shown as mean ± SD. *N* = 4,  ^*∗*^*p* < 0.05,  ^*∗∗*^*p* < 0.01,  ^*∗∗∗*^*p* < 0.005, and  ^*∗∗∗∗*^*p* < 0.001; one-way ANOVA test (c). Result shown is representative of 2-3 independent experiments.

We further determined whether miR-22 contributes to the polarization of macrophages. By controling, miR-22-O/E, or miR-22-Inh RAW 264.7 cells were stimulated with LPS + IFN-*γ* or IL-4 for 24 hr, and the expression of M1 or M2 genes was analyzed. Consistent with the finding that miR-22 overexpression or inhibition reduced or increased TNF production, the expression of pro-inflammatory M1 genes was reduced in miR-22-O/E cells, while miR-22 inhibition increased M1 gene expression (Figure 4(a)). However, IL-4-induced expression of anti-inflammation M2 genes was comparable between control, miR-22-O/E, and miR-22-Inh cells (Figure 4(b)), suggesting that miR-22 negatively regulates the promotion of macrophages to a pro-inflammatory M1 phenotype.

### 3.5. miR-22 Regulates NF-*κ*B and MAPK Pathways in TLR Signaling

Next, we investigated whether the activation of signaling pathways that play a crucial role in pro-inflammatory responses in macrophages was affected by miR-22. Control or miR-22-O/E macrophages were stimulated with LPS, and the activation of NF-*κ*B and MAPK signaling was analyzed. Compared with control cells, the degradation of I*κ*B-*α*, which is an indication of NF-*κ*B activation, and the phosphorylation of p38, ERK, and JNK were significantly reduced or delayed in miR-22-O/E cells (Figure 4(c)), indicating that miR-22 negatively regulates downstream signaling in the TLR pathway, which is essential for the expression of pro-inflammation genes in macrophages.

### 3.6. Regulation of Macrophage Metabolism by miR-22

**Figure 5:**
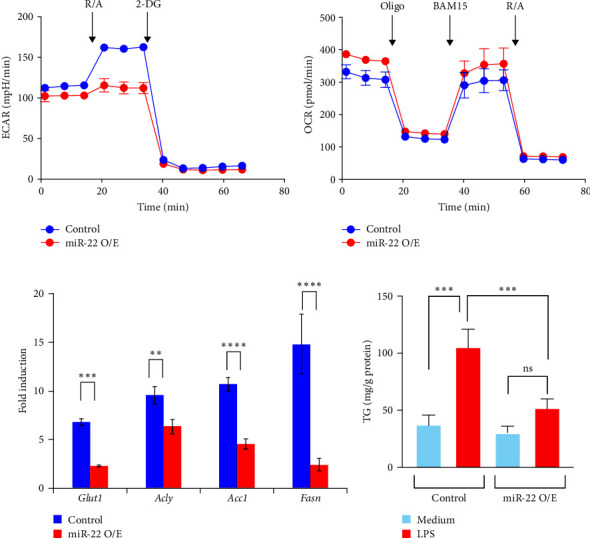
Regulation of cell metabolism by miR-22. (a and b) Glycolysis (a) and mitochondrial respiration (b) were measured in LPS + IFN-*γ*- (a) or IL-4 (b)-stimulated control or miR-22-O/E RAW 264.7 cells on a seahorse XFe96 extracellular flux analyzer (*n* = 6). (c) Expression of metabolism genes was analyzed by qPCR. (d) Control or miR-22-O/E RAW 264.7 cells were treated with LPS for 24 hr and intracellular TG levels were determined. Total protein concentration was measured. *N* = 4. Data are shown as mean ± SD.  ^*∗∗*^*p* < 0.01,  ^*∗∗∗*^*p* < 0.005, and  ^*∗∗∗∗*^*p* < 0.001. One-way ANOVA test. Result shown is representative of 2-3 independent experiments.

Cell metabolism is closely associated with macrophage phenotype determination. M1 macrophages rely on glycolysis and fatty acid synthesis for the expression of pro-inflammatory genes, while M2 macrophages require oxidative phosphorylation and fatty acid oxidation for anti-inflammation gene expression [35–37]. Since we found that miR-22 plays a role in the M1 polarization of macrophages, we further examined whether macrophage metabolism was altered by miR-22. Our real-time metabolism analysis revealed that LPS + IFN-*γ* treatment significantly increased ECAR levels in control macrophages, whereas miR-22 overexpression reduced glycolytic activity (Figure 5(a)). In contrast, IL-4-induced mitochondrial metabolism was comparable between control and miR-22 O/E macrophages (Figure 5(b)). Collectively, our data support a role for miR-22 in regulating the M1 polarization of macrophages, which is associated with metabolism.

It is evident that pro-inflammatory macrophages rely on glycolysis and fatty acid synthesis for the expression of pro-inflammatory genes [35–37]. Thus, we further examined the expression of genes that are related to glycolysis and fatty acid synthesis in macrophages. ATP-citrate lyase (ACLY) is an essential enzyme that catalyzes the critical substrate acetyl-CoA for fatty acid synthesis. ACLY cleaves citrate to acetyl-CoA and oxaloacetate in the presence of ATP and CoA, and acetyl-CoA is then catalyzed by acetyl-CoA carboxylase (ACC), leading to fatty acid synthesis by fatty acid synthase (FASN) [38, 39]. Stimulation of macrophages with LPS induced the expression of *Acly*, *Acc1*, and *Fasn*, which was significantly reduced by miR-22 overexpression. Since these enzymes are not the direct targets of miR-22 in macrophages, it is suggested that the reduced glycolytic activity caused by miR-22 further reduced the expression of genes for fatty acid synthesis (Figure 5(c)). Indeed, intracellular triglyceride (TG) level was increased in LPS-treated control macrophages compared with unstimulated cells; however, overexpression of miR-22 reduced TG level in LPS-treated macrophages (Figure 5(d)), supporting our finding that expression of fatty acid synthesis genes is closely related to the miR-22 activity in macrophages.

In conclusion, our findings demonstrate that miR-22 is induced in TLR-dependent signaling to regulate the expression of GLUT1 and 4-1BBL, which are involved in cell metabolism as well as the pro-inflammatory responses and M1 polarization of macrophages.

## 4. Discussion

miRNAs play a critical role in regulating gene expression. Many miRNAs are selectively expressed in mammalian immune cells, and some have been linked to immune responses in host defense and autoimmune disease by regulating the expression of signaling components [13–17]. MiRNAs regulate pro- or anti-inflammatory macrophage responses, implicating miRNAs as key regulators of inflammation either by promoting or suppressing inflammation [13, 18, 40]. Although novel miRNAs continue to be identified, the biological roles of many in the regulation of macrophage metabolism for inflammatory phenotype determination have not been fully elucidated yet.

To identify miRNAs that regulate the inflammatory response in macrophages, we screened a miRNA library composed of ∼ 650 miRNAs using a lentivirus expression system (Biosettia). After the infection of macrophages with miRNA-encoding lentivirus, cells were treated with LPS for 24 hr. Production of TNF was measured by ELISA and calculated as the percentage of TNF production in miRNA overexpressing cells compared to control miRNA-expressing cells, and some miRNAs increased the production of TNF while others reduced. Among the candidate miRNAs, we found that miR-22 negatively regulates the production of TNF in macrophages (data not shown).

In this study, we investigated the regulatory mechanism of inflammatory responses and phenotype determination in which miR-22 regulates the expression of GLUT1 and 4-1BBL that play a crucial role in glycolysis and sustained inflammation, respectively [41–44].

In our previous study, we discovered that 4-1BBL regulates the TLR-induced sustained inflammatory response of innate immune cells. Production of cytokines such as TNF and IL-23 in vitro by WT mouse macrophages was sustained for 24 hr, while 4-1BBL knockout (KO) macrophages produced cytokines similar to WT cells for the first few hours after LPS treatment but then ceased production. Furthermore, 4-1BBL contributes to the development of inflammatory diseases since ablation of 4-1BBL or inhibition of 4-1BBL signaling significantly reduces cytokine production by macrophages and ameliorates the pathology of mouse models of inflammatory diseases [44–47]. Additionally, emerging evidence suggests that Glut1-mediated glucose metabolism drives pro-inflammatory responses in macrophages [41, 42]. Also, in our study, I knocked down Glut1 by siRNA or inhibited Glut1 activity by 2-deoxyglucose and confirmed the role of glycolysis in macrophage activation [44].

Induction of miR-22 was initiated by TLR signaling and dependent on TLR-proximal signaling pathways such as NF-*κ*B and MAPKs. Overexpression of miR-22, which is similar to the transfection of miR-22 mimics, reduced the expression and production of pro-inflammation genes while inhibition of miR-22 increased the expression, indicating that miR-22 negatively regulates the induction of pro-inflammatory responses in macrophages. Furthermore, we discovered that miR-22 increases the M1 polarization of macrophages as the expression of M1 markers such as *Tnf*, *Il6*, and *Nos2* was increased by miR-22 inhibition while decreased by miR-22 overexpression. However, miR-22 had no effect on the polarization of M2 macrophages, indicating the specific role of miR-22 in regulating the pro-inflammatory responses of macrophages.

Despite a body of evidence that suggests a role of miRNAs for the regulation of macrophage activation, polarization, and cell metabolism [19–21], critical questions remain concerning the molecular basis underlying the mechanism of targeting cell metabolism. Using bioinformatics tools, we initiated the search for candidate pairings and found that 4-1BBL, a member of the TNF superfamily, and GLUT1 are the targets of miR-22. Previous studies suggested that activated macrophages rely on glycolysis for ATP generation, which is similar to the cellular events in cancer cells called the Warburg effect [48–50]. GLUT1-mediated glycolysis drives a pro-inflammatory phenotype of M1 macrophages [41–44], supporting the role of miR-22 in the regulation of GLUT1.

Collectively, our data indicate that activation of TLR signaling quickly initiated the expression of *Slc2a1* and *tnfsf9* for the inflammatory responses in macrophages, which was then followed by the induction of miR-22, leading to the downregulation of *Slc2a1* and *tnfsf9* and the reduction of TNF production at the late phase of macrophage activation, i.e., after 8–12 hr of TLR stimulation. This is reminiscent of 4-1BBL-deficient macrophages [45, 46], suggesting the negative regulation of 4-1BBL and GLUT1 by miR-22 for the downregulation of sustained inflammation in macrophages and also implicating that miR-22-mediated targeting of inflammatory responses of macrophages can be an option for treating inflammatory disease.

Previously, we identified a novel mechanism that is mediated by 4-1BBL that regulates sustained inflammation in macrophages [45, 46]. Deletion of 4-1BBL reduced the expression of pro-inflammatory cytokines and M1 polarization markers in LPS/IFN-*γ*-treated macrophages [44], which is consistent with the results of miR-22-O/E cells. Furthermore, we discovered that cell metabolism regulates TLR-mediated 4-1BBL expression, as inhibition or knockdown of glycolysis or fatty acid synthesis reduced 4-1BBL expression, and 4-1BBL-mediated inflammation is dependent on glycolysis and fatty acid synthesis [44].

A previous study also showed that miR-22 is upregulated in mature dendritic cells and regulates the induction of *Csf1r* mRNA [51]. This result supports our finding that miR-22 negatively regulates the inflammatory responses of macrophages although miR-22 targets different gene expression. In contrast, it is also reported that treatment of LPS downregulates the levels of miR-22 in macrophages leading to the increased expression of histone deacetylase 6 (*HDAC6*) for the activation of NF-*κ*B and AP-1, resulting in the induction of inflammatory responses [52]. Collectively, it is suggested that miR-22 regulates the induction of different target genes that contribute to the regulation of inflammatory responses of macrophages.

Consistent with the role of miR-22 in M1 macrophage polarization, the real-time metabolism analysis showed that miR-22 regulates the glycolytic activity of macrophages and contributes to the metabolic reprograming of macrophages. However, IL-4-mediated mitochondrial respiration, which is a hallmark of M2 polarization, was unaffected by miR-22 overexpression. Furthermore, the reduction of *Slc2a1* by miR-22 led to a decrease in fatty acid synthesis as TG accumulation induced by TLR4 activation [53] was significantly lower in miR-22 O/E macrophages compared with control cells. Collectively, our data suggest that miR-22 regulates the activation and metabolic reprograming of macrophages by targeting *Slc2a1* and *tnfsf9* which play a regulatory role in pro-inflammatory responses.

It has been demonstrated that 4-1BBL and GLUT1 contribute to the pathology of psoriasis in mice [44, 54]. Thus, we investigated whether miR-22 may play a role in skin inflammation by regulating the expression of *Slc2a1* and *tnfsf9* in macrophages. In the imiquimod (IMQ)-induced psoriasis-like skin inflammation model in mice, IMQ treatment on the shaved dorsal skin induces skin inflammation that is similar to the pathology of psoriasis in humans. Expression of *tnfsf9* and *Slc2a1* in immune cells was induced early by IMQ treatment and reduced thereafter in mice [44], which is consistent with the changes in the severity of skin inflammation. We found that miR-22 was first detected in skin macrophages on Day 2 after IMQ treatment and that miR-22 levels peaked on Day 4 before declining. In contrast, miR-22 induction was detected at Day 4 in skin *T* cells, although the miR-22 level in *T* cells was lower than that in macrophages. MiR-22 induction, however, was not detected in keratinocytes of IMQ-treated mice (*Supplementary 2*). This data implicates that 4-1BBL and GLUT1 are expressed in IMQ-induced skin inflammation, followed by the induction of miR-22 to downregulate their expression levels for the spontaneous reduction of skin inflammation in mice. Since TNF is involved in some autoimmune and inflammatory diseases [55, 56], our result implicates the therapeutic potential of miR-22 for psoriasis treatment by targeting the production of TNF.

In conclusion, we have demonstrated a novel role of miR-22 in the regulation of macrophage metabolism and phenotype determination. TLR-dependent induction of miR-22 regulates the expression of *Slc2a1* and *tnfsf9* mRNAs that contribute to the sustained inflammatory responses in macrophages, thereby regulating the inflammatory responses and metabolic reprograming associated with macrophage phenotype determination. Our observations support further exploration of potential therapeutic strategies using miR-22 for the modulation of macrophage functional responses in inflammatory disease development.

## 5. Conclusion

Activation of TLR signaling induces the expression of genes that play a crucial role in the sustained inflammatory responses in macrophages. MiR-22, one of the miRNAs that are induced by TLR stimulation, is induced after the expression of its target genes such as *Slc2a1* and *Tnfsf9*, and downregulates their expression for the resolution of excessive inflammatory responses in macrophages. Therefore, a strategy using miR-22 can be an option for the treatment of inflammatory diseases by reducing the inflammatory responses of macrophages.

## Data Availability

The data that support the findings of this study are available from the corresponding author upon reasonable request.

## Abbreviations

miRNA: MicroRNA
GLUT1: Glucose transporter 1
UTR: Untranslated region
TNFSF9: Tumor necrosis factor 9.

## Supplementary Materials

Supplementary 1 Primer sequences for quantitative PCR.

Supplementary 2 Induction of miR-22 in immune cells and keratinocytes in IMQ-treated mice.
