# Supplementary material for: MicroRNA-22 Regulates the Pro-inflammatory Responses and M1 Polarization of Macrophages by Targeting GLUT1 and 4-1BBL
Source: J Immunol Res. 2023 Jul 10;2023:2457006. doi: 10.1155/2023/2457006 (PMC10352528; doi:10.1155/2023/2457006)
Supplement: Supplementary 1 — Primer sequences for quantitative PCR. [file 2457006.f1.docx]

Supplementary Table 1. Primer sequences for quantitative PCR.

| Gene | Forward (5’→ 3’) | Reverse (5’→ 3’) |
| --- | --- | --- |
| *Il6* | GAGGATACCACTCCCAACAGACC | AAGTGCATCATCGTTGTTCATACA |
| *Il10* | TGGCCCAGAAATCAAGGAGC | CAGCAGACTCAATACACACT |
| *Il23* | GGTGGCTCAGGGAAATGT | GACAGAGCAGGCAGGTACAG |
| *Tgfb* | CAACGCCATCTATGAGAAAACC | AAGCCCTGTATTCCGTCTCC |
| *Tnf* | ATGAGCACAGAAAGCATGA | AGTAGACAGAAGAGCGTGGT |
| *Tnfsf9* | ATTCACAAACACAGGCCACA | CTCCAGGAACGGTCCACTAA |
| *Nos2* | CCCTTCCGAAGTTTCTGGCAGCA | GGCTGTCAGAGAGCCTCGTGGCTTT |
| *Arg1* | CCAGAAGAATGGAAGAGTCAGTGT | GCAGATATGCAGGGAGTCACC |
| *Cxcl10* | ATGACGGGCCAGTGAGAATG | GAGGCTCTCTGCTGTCCATC |
| *Fizz1* | GGAACTTCTTGCCAATCCAG | AGCACACCCAGTAGCAGTCA |
| *Ym1* | ACCCCTGCCTGTGTACTCACCT | CACTGAACGGGGCAGGTCCAAA |
| *Egr2* | TGCTAGCCCTTTCCGTTGA | TCTTTTCCGCTGTCCTCGAT |
| *Mrc1* | CTCTGTTCAGCTATTGGACGC | CGGAATTTCTGGGATTCAGCTTC |
| *Slc2a1* | GTCGCCTCATTCTTTGGTG | CTGATACACTTCGTCCAGC |
| *Acaca* | ATTGTGGCTCAAACTGCAGGT | GCCAATCCACTCGAAGACCA |
| *Acly* | AGGAAGTGCCACCTCCAACAGT | CGCTCATCACAGATGCTGGTCA |
| *Gapdh* | TCAAGAAGGTGGTGAAGCAG | TCGCTGTTGAAGTCAGAGGA |
| *Fasn* | TTGCTGGCACTACAGAATGC | AACAGCCTCAGAGCGACAAT |
