# Supplementary material for: MicroRNA-22 Regulates the Pro-inflammatory Responses and M1 Polarization of Macrophages by Targeting GLUT1 and 4-1BBL
Source: J Immunol Res. 2023 Jul 10;2023:2457006. doi: 10.1155/2023/2457006 (PMC10352528; doi:10.1155/2023/2457006)
Supplement: Supplementary 2 — Induction of miR-22 in immune cells and keratinocytes in IMQ-treated mice. [file 2457006.f2.pdf]

Supplementary Figure 1

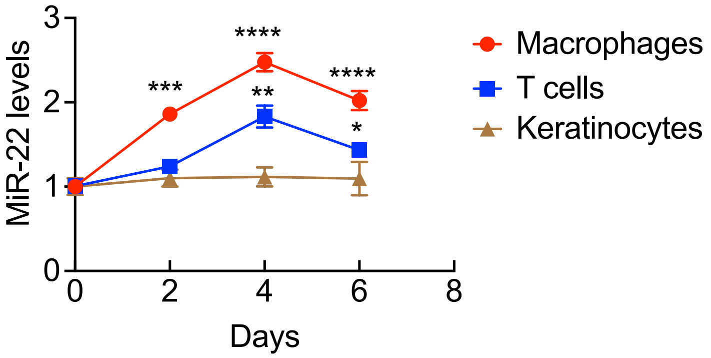

Supplementary Figure 1. Induction of miR-22 in immune cells and keratinocytes in IMQ-treated mice. Macrophages, T cells and keratinocytes were prepared from the skin tissues of IMQ-treated mice at the indicated times, and the levels of miR-22 was measured by qPCR analysis. Data are shown as mean ± SD. N = 3, *$p < 0.05$, **$p < 0.01$, ***$p < 0.005$, ****$p < 0.001$. RNA samples were collected and analyzed in our previous study (Miki, H. et al. *J Immunol.* **204**, 1892-1903 (2020).
